# On some Hermite–Hadamard type inequalities for (*s*, QC)-convex functions

**DOI:** 10.1186/s40064-016-1676-9

**Published:** 2016-01-20

**Authors:** Ying Wu, Feng Qi

**Affiliations:** College of Mathematics, Inner Mongolia University for Nationalities, Tongliao City, 028043 Inner Mongolia Autonomous Region China; Department of Mathematics, School of Science, Tianjin Polytechnic University, Tianjin City, 300160 China; Institute of Mathematics, Henan Polytechnic University, Jiaozuo City, 454010 Henan Province China

**Keywords:** Convex function, (*s*, QC)-Convex function on the co-ordinates, Hermite–Hadamard’s integral inequality, 26A51, 26D15, 26D20, 26E60, 41A55

## Abstract

In the paper, the authors introduce a new notion “$(s,\text {QC})$-convex function on the co-ordinates” and establish some Hermite–Hadamard type integral inequalities for $(s,\text {QC})$-convex functions on the co-ordinates.

## Background

Let $f:I\subseteq \mathbb {R}\rightarrow \mathbb {R}$ be a convex function and $a, b\in I$ with $a < b$. The double inequality

1 $$\begin{aligned} f\biggl (\frac{a+b}{2}\biggr ) \le \frac{1}{b-a} \int _{a}^{b}f(x)\mathrm{d}x \le \frac{f(a)+f(b)}{2} \end{aligned}$$

is known in the literature as Hermite–Hadamard’s inequality for convex functions.

### **Definition 1**

(Dragomir and Pearce 1998; Pečarić et al. 1992) A function $f:I\subseteq \mathbb {R}\rightarrow \mathbb {R}$ is said to be quasi-convex (QC), if

2 $$\begin{aligned} f(\lambda x+(1-\lambda )y)\le \max \{f(x),f(y)\} \end{aligned}$$

holds for all $x,y\in I$ and $\lambda \in [0,1]$.

### **Definition 2**

(Dragomir and Pearce 1998) The function $f:I\subseteq \mathbb {R}\rightarrow \mathbb {R}$ is Jensen- or J-quasi-convex (JQC) if

3 $$\begin{aligned} f\biggl (\frac{x+y}{2}\biggl )\le \max \{f(x),f(y)\} \end{aligned}$$

holds for all $x,y\in I$.

### **Definition 3**

(Hudzik and Maligranda 1994) Let $s\in (0, 1]$. A function $f:I\subseteq \mathbb {R}\rightarrow \mathbb {R}$ is said to be *s*-convex (in the second sense) if

4 $$\begin{aligned} f(\lambda x+(1-\lambda )y)\le \lambda ^sf(x)+(1-\lambda )^sf(y) \end{aligned}$$

holds for all $x,y\in I$ and $\lambda \in [0,1].$

### **Definition 4**

(Xi and Qi 2015a) For some $s\in [-1,1]$, a function $f:I\subseteq \mathbb {R}\rightarrow \mathbb {R}$ is said to be extended *s*-convex if

5 $$\begin{aligned} f(\lambda x+(1-\lambda )y)\le \lambda ^sf(x)+(1-\lambda )^sf(y) \end{aligned}$$

is valid for all $x,y\in I$ and $\lambda \in (0,1)$.

### **Definition 5**

(Dragomir 2001; Dragomir and Pearce 2000) A function $f:\Delta =[a,b]\times [c,d]\subseteq \mathbb {R}^2 \rightarrow \mathbb {R}$ is said to be convex on co-ordinates on $\Delta$ if the partial functions

6 $$\begin{aligned} f_y:[a,b]\rightarrow \mathbb {R},\quad f_y(u)=f_y(u,y)\quad \text {and}\quad f_x:[c,d]\rightarrow \mathbb {R},\quad f_x(v)= f_x(x,v) \end{aligned}$$

are convex for all $x\in (a,b)$ and $y \in (c,d)$.

### **Definition 6**

A function $f:\Delta =[a,b]\times [c,d]\subseteq \mathbb {R}^2 \rightarrow \mathbb {R}$ is said to be convex on co-ordinates on $\Delta$ if the inequality

7 $$\begin{aligned}&f(tx+(1-t)z,\lambda y+(1-\lambda )w)\nonumber \\&\quad \le t\lambda f(x,y)+t(1-\lambda )f(x,w)+(1-t)\lambda f(z,y)+(1-t)(1-\lambda )f(z,w) \end{aligned}$$

holds for all $t,\lambda \in [0,1]$ and $(x,y),(z,w)\in \Delta$.

### **Definition 7**

(Alomari and Darus 2008) A function $f:\Delta =[a,b]\times [c,d]\subseteq \mathbb {R}^2 \rightarrow \mathbb {R}_0=[0,\infty )$ is *s*-convex on $\Delta$ for some fixed $s\in (0, 1]$ if

8 $$\begin{aligned} f(\lambda x + (1-\lambda )z, \lambda y + (1-\lambda )w)\le \lambda ^sf(x, y)+(1-\lambda )^sf(z,w) \end{aligned}$$

holds for all $(x, y), (z,w)\in \Delta$ and $\lambda \in [0, 1]$.

### **Definition 8**

(Özdemir et al. 2012a, Definition 7) A function $f:\Delta =[a,b]\times [c,d]\subseteq \mathbb {R}^2 \rightarrow \mathbb {R}$ is called a Jensen- or J-quasi-convex function on the co-ordinates on $\Delta$ if

9 $$\begin{aligned} f\left( \frac{x +z}{2},\frac{y +w}{2}\right) \le \max \{f(x, y), f(z,w)\} \end{aligned}$$

holds for all $(x, y), (z,w)\in \Delta$.

### **Definition 9**

(Özdemir et al. 2012a, Definition 5) A function $f:\Delta =[a,b]\times [c,d]\subseteq \mathbb {R}^2 \rightarrow \mathbb {R}$ is called a quasi-convex function on the co-ordinates on $\Delta$ if

10 $$\begin{aligned} f(\lambda x + (1-\lambda )z, \lambda y + (1-\lambda )w)\le \max \{f(x, y), f(z,w)\} \end{aligned}$$

holds for all $(x, y), (z,w)\in \Delta$ and $\lambda \in [0, 1]$.

### **Theorem 1**

(Dragomir 2001; Dragomir and Pearce 2000 Theorem 2.2) *Let*$f:\Delta =[a,b]\times [c,d]\subseteq \mathbb {R}^2 \rightarrow \mathbb {R}$*be convex on the co-ordinates on*$\Delta$*with*$a<b$*and*$c<d$. *Then*

$$\begin{aligned}&f\biggl (\frac{a+b}{2},\frac{c + d}{2}\biggr )\\&\quad \le \frac{1}{2}\biggl [ \frac{1}{b - a}\int _a^b f\biggl (x,\frac{c + d}{2}\biggr )\mathrm{d}x + \frac{1}{d -c}\int _c^df\biggl (\frac{a + b}{2},y\biggr ) \mathrm{d}y\biggr ]\\&\quad \le \frac{1}{(b - a)(d - c)}\int _a^b \int _c^d f(x,y)\mathrm{d}y\mathrm{d}x\\&\quad \le \frac{1}{4}\biggl [\frac{1}{b - a}\biggl (\int _a^b f(x,c)\mathrm{d}x + \int _a^bf(x,d)\mathrm{d}x\biggr ) + \frac{1}{d - c}\biggl (\int _c^d f(a,y) \mathrm{d}y + \int _c^df(b,y)\mathrm{d}y\biggr )\biggr ]\\&\quad \le \frac{f(a,c) + f(b,c) + f(a,d) + f(b,d)}{4}. \end{aligned}$$

### **Theorem 2**

(Özdemir et al. 2012a, Lemma 8) *Every J-quasi-convex mapping*$f:\Delta =[a,b]\times [c,d]\subseteq \mathbb {R}^2 \rightarrow \mathbb {R}$*is J-quasi-convex on the co-ordinates*.

### **Theorem 3**

(Özdemir et al. 2012a, Lemma 6) *Every quasi-convex mapping*$f:\Delta =[a,b]\times [c,d]\subseteq \mathbb {R}^2 \rightarrow \mathbb {R}$*is quasi-convex on the coordinates*.

For more information on this topic, please refer to Bai et al. (2016), Hwang et al. (2007), Özdemir et al. (2011, 2012a, b, c, 2014), Qi and Xi (2013), Roberts and Varberg (1973), Sarikaya et al. (2012), Wu et al. (2016), Xi et al. (2012, 2015), Xi and Qi (2012, 2013, 2015a, b, c) and related references therein.

In this paper, we introduce a new concept “$(s, \text {QC})$-convex functions on the co-ordinates on the rectangle of $\mathbb {R}^2$” and establish some new integral inequalities of Hermite–Hadamard type for $(s, \text {QC})$-convex functions on the co-ordinates.

## Definitions and Lemmas

We now introduce three new definitions

### **Definition 10**

For $s \in [-1,1]$, a function $f:\Delta =[a,b]\times [c,d]\subseteq \mathbb {R}^2 \rightarrow \mathbb {R}_0$ is said to be $(\text {J}s, \text {JQC})$-convex on the co-ordinates on $\Delta$ with $a<b$ and $c<d$, if

11 $$\begin{aligned} f\biggl (\frac{x+z}{2},\frac{y+w}{2}\biggr ) \le \frac{1}{2^{s}}\bigl [\max \{f(x,y),f(x,w)\} +\max \{f(z,y),f(z,w)\}\bigr ] \end{aligned}$$

holds for all $t, \lambda \in [0,1]$ and $(x,y),(z,w)\in \Delta$.

### *Remark 1*

By Definitions 8 and 10 and Lemma 1, we see that, for $s \in [-1,1]$ and $f:\Delta \subseteq \mathbb {R}^2 \rightarrow \mathbb {R}_0$,

1. If $f:\Delta \rightarrow \mathbb {R}_0$ is a J-quasi-convex function on the co-ordinates on $\Delta$, then *f* is a $(\text {J}s, \text {JQC})$-convex function on the co-ordinates on $\Delta$;
2. Every J-quasi-convex function $f:\Delta \rightarrow \mathbb {R}_0$ is a $(\text {J}s, \text {JQC})$-convex function on the co-ordinates on $\Delta$.

### **Definition 11**

A function $f:\Delta =[a,b]\times [c,d]\subseteq \mathbb {R}^2\rightarrow \mathbb {R}_0$ is called $(s, \text {JQC})$-convex on the co-ordinates on $\Delta$ with $a<b$ and $c<d$, if

12 $$\begin{aligned} f\biggl (tx+(1-t)z,\frac{y+w}{2}\biggr ) \le t^s\max \{f(x,y),f(x,w)\} +(1-t)^s\max \{f(z,y),f(z,w)\} \end{aligned}$$

holds for all $t\in (0,1)$, $(x,y),(z,w) \in \Delta$, and some $s \in [-1,1]$.

### **Definition 12**

For some $s \in [-1,1]$, a function $f:\Delta =[a,b]\times [c,d]\subseteq \mathbb {R}^2\rightarrow \mathbb {R}_0$ is called $(s, \text {QC})$-convex on the co-ordinates on $\Delta$ with $a<b$ and $c<d$, if

13 $$\begin{aligned}&f(tx+(1-t)z,\lambda y+(1-\lambda )w)\nonumber \\&\quad \le t^s\max \{f(x,y),f(x,w)\} +(1-t)^s\max \{f(z,y),f(z,w)\} \end{aligned}$$

is valid for all $t\in (0,1)$, $\lambda \in [0,1]$, and $(x,y),(z,w) \in \Delta$.

### *Remark 2*

For $$s \in (0,1]$$ and $$f:\Delta \subseteq \mathbb {R}^2 \rightarrow \mathbb {R}_0$$,

1. If taking $\lambda =\frac{1}{2}$ and $t=\lambda =\frac{1}{2}$ in (13), then $(\text {J}s,\text {JQC})\subseteq (s, \text {JQC})\subseteq (s, \text {QC})$;
2. If $f:\Delta \rightarrow \mathbb {R}_0$ is a *s*-convex function on $\Delta$, then *f* is an $(s, \text {QC})$-convex function on the co-ordinates on $\Delta$.

### *Remark 3*

Considering Definitions 9 and 12 and Lemma 1, for $s \in [-1,1]$ and $f:\Delta \subseteq \mathbb {R}^2 \rightarrow \mathbb {R}_0$,

1. If $f:\Delta \rightarrow \mathbb {R}_0$ is a quasi-convex function on the co-ordinates on $\Delta$, then it is an $(s, \text {QC})$-convex function on the co-ordinates on $\Delta$;
2. Every quasi-convex function $f:\Delta \rightarrow \mathbb {R}_0$ is an $(s, \text {QC})$-convex function on the co-ordinates on $\Delta$.

### **Lemma 1**

(Latif and Dragomir 2012) *If*$f:\Delta =[a,b]\times [c,d]\subseteq \mathbb {R}^2 \rightarrow \mathbb {R}$*has partial derivatives and*$\frac{\partial ^2f}{\partial x\partial y}\in L_1(\Delta )$*with*$a<b$*and*$c<d$, *then*

$$\begin{aligned} \Phi (f;a,b,c,d)\triangleq&\frac{1}{(b-a)(d-c)}\int _a^b\int _c^df(x,y)\mathrm{d}y\mathrm{d}x+f\left( \frac{a+b}{2},\frac{c+d}{2}\right) \\&-\frac{1}{b-a}\int _a^bf\biggl (x,\frac{c+d}{2}\biggr )\mathrm{d}x -\frac{1}{d-c}\int _c^df\biggl (\frac{a+b}{2},y\biggr ) \mathrm{d}y\\ &=(b-a)(d-c)\int _0^1 \int _0^1 K(t,\lambda ) \frac{\partial ^2}{\partial x\partial y}f(ta+(1 - t)b,\lambda c+(1-\lambda )d)\mathrm{d}t\mathrm{d}\lambda , \end{aligned}$$

*where*

14 $$\begin{aligned} K(t,\lambda )= {\left\{ \begin{array}{ll} t\lambda , &{}(t,\lambda ) \in \bigr [0,\frac{1}{2}\bigr ]\times \bigr [0,\frac{1}{2}\bigr ], \\ t\bigr (\lambda - 1), &{}(t,\lambda ) \in \bigr [0,\frac{1}{2}\bigr ]\times \bigr (\frac{1}{2},1\bigr ], \\ \bigr (t - 1)\lambda , &{}(t,\lambda ) \in \bigr (\frac{1}{2},1\bigr ]\times \bigr [0,\frac{1}{2}\bigr ], \\ \bigr (t - 1)\bigr (\lambda - 1),&{}(t,\lambda ) \in \bigr (\frac{1}{2},1\bigr ]\times \bigr (\frac{1}{2},1\bigr ]. \end{array}\right. } \end{aligned}$$

### **Lemma 2**

*Let*$r\ge 0$*and*$q>1$. *Then*

15 $$\begin{aligned} \int _0^{1/2}u^{r}\mathrm{d}u&=\int _{1/2}^1(1-u )^{r}\mathrm{d}u =\frac{1}{2^{r+1}(r+1)} \end{aligned}$$

*and*

16 $$\begin{aligned} \int _0^1\int _0^1|K(t,\lambda )|^{q/(q-1)}\mathrm{d}t\mathrm{d}\lambda =\biggl (\frac{q-1}{2q-1}\biggl )^2\biggl (\frac{1}{4}\biggr )^{q/(q-1)}. \end{aligned}$$

*where*$K(t,\lambda )$*is defined by* (14).

### *Proof*

This follows from a straightforward computation.$\square$

## Some integral inequalities of Hermite–Hadamard type

In this section, we will establish Hermite–Hadamard type integral inequalities for $(s, \text {QC})$-convex functions on the co-ordinates on rectangle from the plane $\mathbb {R}^2$.

### **Theorem 4**

*Let*$f:\Delta =[a,b]\times [c,d]\subseteq \mathbb {R}^2\rightarrow \mathbb {R}$*have partial derivatives and*$\frac{\partial ^2f}{\partial x\partial y}\in L_1(\Delta )$. *If*$\bigl | \frac{\partial ^2f}{\partial x\partial y}\bigr |^q$*is an* (*s*, QC)-*convex function on the co-ordinates on*$\Delta$*with*$a<b$*and*$c<d$*for some*$s \in [-1,1]$*and*$q\ge 1$, *then*

1. *When*$$s \in (-1,1]$$,
  17 $$\begin{aligned} \begin{array}{ll} |\Phi (f;a,b,c,d)| &{}\le \frac{(b-a)(d-c)}{8}\biggl (\frac{1}{2^{s+1}(s+1)(s+2)}\biggr )^{1/q}\\ &{}\quad \times \Bigl \{\bigl [(s+1)M_q(a,c,d)+(2^{s+2}-s-3)M_q(b,c,d)\bigl ]^{1/q}\\ &{}\quad +\bigl [(2^{s+2}-s-3)M_q(a,c,d) +(s+1)M_q(b,c,d)\bigl ]^{1/q}\Bigl \}; \end{array} \end{aligned}$$
2. *When*$$s=-1$$,
  18 $$\begin{aligned} |\Phi (f;a,b,c,d)|\le & {} \frac{(b-a)(d-c)}{8}\Bigl \{\bigl [M_q(a,c,d)+(2\ln 2-1)M_q(b,c,d)\bigl ]^{1/q}\nonumber \\&+\bigl [(2\ln 2-1)M_q(a,c,d)+M_q(b,c,d)\bigl ]^{1/q}\Bigl \}; \end{aligned}$$

*where*

19 $$\begin{aligned} M_q(u,c,d)=\max \biggl \{\biggl |\frac{\partial ^2}{\partial x\partial y}f(u,c)\biggl |^q, \biggl | \frac{\partial ^2}{\partial x\partial y}f(u,d)\biggl |^q\biggl \}. \end{aligned}$$

### *Proof*

By Lemma 1 and Hölder’s integral inequality, we have

20 $$\begin{aligned} \begin{array}{ll} |\Phi &{}(f;a,b,c,d)| \\ &{}\le (b-a)(d-c)\int _0^1\int _0^1|K(t,\lambda )| \biggl |\frac{\partial ^2}{\partial x\partial y}f(ta+(1-t)b, \lambda c+(1-\lambda )d)\biggr |\mathrm{d}t\mathrm{d}\lambda \\ &{}\le (b-a)(d-c)\biggl (\int _0^{1}\int _0^{1}|K(t,\lambda )|\mathrm{d}t\mathrm{d}\lambda \biggr )^{1-1/q}\\ &{}\quad \times \biggl \{ \biggl [\int _0^{1/2}\int _0^{1/2}t\lambda \biggl | \frac{\partial ^2}{\partial x\partial y} f(ta+(1-t)b,\lambda c+(1-\lambda )d)\biggr |^q\mathrm{d}t\mathrm{d}\lambda \biggl ]^{1/q}\\ &{}\quad +\biggl [\int _{1/2}^1\int _0^{1/2}t(1-\lambda )\biggl | \frac{\partial ^2}{\partial x\partial y} f(ta+(1-t)b,\lambda c+(1-\lambda )d)\biggr |^q\mathrm{d}t\mathrm{d}\lambda \biggl ]^{1/q}\\ &{}\quad +\biggl [\int _0^{1/2}\int _{1/2}^1(1-t)\lambda \biggl | \frac{\partial ^2}{\partial x\partial y} f(ta+(1-t)b,\lambda c+(1-\lambda )d)\biggr |^q\mathrm{d}t\mathrm{d}\lambda \biggl ]^{1/q}\\ &{}\quad +\biggl [ \int _{1/2}^1\int _{1/2}^1(1-t)(1-\lambda )\biggl | \frac{\partial ^2}{\partial x\partial y} f(ta+(1-t)b,\lambda c+(1-\lambda )d)\biggr |^q\mathrm{d}t\mathrm{d}\lambda \biggl ]^{1/q}\biggl \}. \end{array} \end{aligned}$$

When $s \in (-1,1]$, using the co-ordinated $(s, \text {QC})$-convexity of $\bigl | \frac{\partial ^2f}{\partial x\partial y}\bigr |^q$ and by Lemma 2, we obtain

21 $$\begin{aligned} \begin{array}{ll} \int _0^{1/2}&{}\int _0^{1/2}t\lambda \biggl | \frac{\partial ^2}{\partial x\partial y} f(ta+(1-t)b,\lambda c+(1-\lambda )d)\biggr |^q\mathrm{d}t\mathrm{d}\lambda \\ &{}\le \biggl (\int _0^{1/2}\lambda \mathrm{d}\lambda \biggr )\int _0^{1/2} \biggl [t^{s+1}\max \biggl \{\biggl |\frac{\partial ^2}{\partial x\partial y}f(a,c)\biggl |^q, \biggl | \frac{\partial ^2}{\partial x\partial y}f(a,d)\biggl |^q\biggl \}\\ &{}\quad +t(1-t)^{s}\max \biggl \{\biggl |\frac{\partial ^2}{\partial x\partial y}f(b,c)\biggl |^q, \biggl | \frac{\partial ^2}{\partial x\partial y}f(b,d)\biggl |^q\biggl \} \biggr ]\mathrm{d}t\\ &{}=\frac{1}{2^{s+5}(s+1)(s+2)}\bigl [(s+1)M_q(a,c,d) +(2^{s+2}-s-3)M_q(b,c,d)\bigr ]. \end{array} \end{aligned}$$

Similarly, we also have

22 $$\begin{aligned} \begin{array}{ll} &{}\int _{1/2}^1\int _0^{1/2}t(1-\lambda )\biggl | \frac{\partial ^2}{\partial x\partial y} f(ta+(1-t)b,\lambda c+(1-\lambda )d)\biggr |^q\mathrm{d}t\mathrm{d}\lambda \\ &{}\quad \le \frac{1}{2^{s+5}(s+1)(s+2)}\bigl [(s+1)M_q(a,c,d)+ (2^{s+2}-s-3)M_q(b,c,d)\bigr ], \end{array} \end{aligned}$$

23 $$\begin{aligned} \begin{array}{ll} &{}\int _0^{1/2}\int _{1/2}^1(1-t)\lambda \biggl | \frac{\partial ^2}{\partial x\partial y} f(ta+(1-t)b,\lambda c+(1-\lambda )d)\biggr |^q\mathrm{d}t\mathrm{d}\lambda \\ &{}\quad \le \frac{1}{2^{s+5}(s+1)(s+2)}\bigl [(2^{s+2}-s-3)M_q(a,c,d) +(s+1)M_q(b,c,d)\bigr ], \end{array} \end{aligned}$$

24 $$\begin{aligned} \begin{array}{ll} &{}\int _{1/2}^1\int _{1/2}^1(1-t)(1-\lambda )\biggl | \frac{\partial ^2}{\partial x\partial y} f(ta+(1-t)b,\lambda c+(1-\lambda )d)\biggr |^q\mathrm{d}t\mathrm{d}\lambda \\ &{}\quad \le \frac{1}{2^{s+5}(s+1)(s+2)}\bigl [(2^{s+2}-s-3)M_q(a,c,d) +(s+1)M_q(b,c,d)\bigr ]. \end{array} \end{aligned}$$

Applying inequalities (21) to (24) into the inequality (20) yields

$$\begin{aligned}&|\Phi (f;a,b,c,d)| \le (b-a)(d-c)\biggl (\frac{1}{16}\biggr )^{1-1/q}\\&\qquad \times \biggl \{ 2\biggl [\frac{1}{2^{s+5}(s+1)(s+2)}\bigl [(s+1)M_q(a,c,d)+(2^{s+2}-s-3)M_q(b,c,d)\biggl ]^{1/q}\\&\qquad +2\biggl [\frac{1}{2^{s+5}(s+1)(s+2)}\bigl [(2^{s+2}-s-3)M_q(a,c,d) +(s+1)M_q(b,c,d)\bigr ]\biggl ]^{1/q}\biggl \}\\&\quad =\frac{(b-a)(d-c)}{8}\biggl (\frac{1}{2^{s+1}(s+1)(s+2)}\biggr )^{1/q}\\&\qquad \times \Bigl \{\bigl [(s+1)M_q(a,c,d)+(2^{s+2}-s-3)M_q(b,c,d)\bigl ]^{1/q}\\&\qquad +\bigl [(2^{s+2}-s-3)M_q(a,c,d) +(s+1)M_q(b,c,d)\bigl ]^{1/q}\Bigl \}. \end{aligned}$$

When $s=-1$, similar to the proof of inequalities (21) to (24), we can write

25 $$\begin{aligned} \begin{array}{ll} &{}\int _0^{1/2}\int _0^{1/2}t\lambda \biggl | \frac{\partial ^2}{\partial x\partial y} f(ta+(1-t)b,\lambda c+(1-\lambda )d)\biggr |^q\mathrm{d}t\mathrm{d}\lambda \\ &{}\quad \le \frac{1}{2^{4}}\bigl [M_q(a,c,d)+(2\ln 2-1)M_q(b,c,d)\bigl ], \end{array} \end{aligned}$$

26 $$\begin{aligned} \begin{array}{ll} &{}\int _{1/2}^1\int _0^{1/2}t(1-\lambda )\biggl | \frac{\partial ^2}{\partial x\partial y} f(ta+(1-t)b,\lambda c+(1-\lambda )d)\biggr |^q\mathrm{d}t\mathrm{d}\lambda \\ &{}\quad \le \frac{1}{2^{4}}\bigl [M_q(a,c,d)+(2\ln 2-1)M_q(b,c,d)\bigl ], \end{array} \end{aligned}$$

27 $$\begin{aligned} \begin{array}{ll} &{}\int _0^{1/2}\int _{1/2}^1(1-t)\lambda \biggl | \frac{\partial ^2}{\partial x\partial y} f(ta+(1-t)b,\lambda c+(1-\lambda )d)\biggr |^q\mathrm{d}t\mathrm{d}\lambda \\ &{}\quad \le \frac{1}{2^{4}}\bigl [(2\ln 2-1)M_q(a,c,d)+M_q(b,c,d)\bigl ], \end{array} \end{aligned}$$

28 $$\begin{aligned} \begin{array}{ll} &{}\int _{1/2}^1\int _{1/2}^1(1-t)(1-\lambda )\biggl | \frac{\partial ^2}{\partial x\partial y} f(ta+(1-t)b,\lambda c+(1-\lambda )d)\biggr |^q\mathrm{d}t\mathrm{d}\lambda \\ &{}\quad \le \frac{1}{2^{4}}\bigl [(2\ln 2-1)M_q(a,c,d)+M_q(b,c,d)\bigl ]. \end{array} \end{aligned}$$

Substituting inequalities (25) to (28) into (20) leads to the inequality (18). Theorem 4 is thus proved.$\square$

### **Corollary 1**

*Under the conditions of Theorem* 4,

1. *If*$q=1$*and*$s \in (-1,1]$, *then*
  $$\begin{aligned} |\Phi (f;a,b,c,d)|\le & {} \frac{(b-a)(d-c)(2^{s+1}-1)}{2^{s+3}(s+1)(s+2)}\\&\times \biggl [\max \biggl \{\biggl | \frac{\partial ^2f(a,c)}{\partial x\partial y}\biggl |, \biggl | \frac{\partial ^2f(a,d)}{\partial x\partial y}\biggl |\biggl \} +\max \biggl \{\biggl | \frac{\partial ^2f(b,c)}{\partial x\partial y}\biggl |, \biggl | \frac{\partial ^2f(b,d)}{\partial x\partial y}\biggl |\biggl \}\biggl ]; \end{aligned}$$
2. *If*$q=1$*and*$s=-1$, *then*
  $$\begin{aligned} |\Phi (f;a,b,c,d)|\le & {} \frac{(b-a)(d-c)\ln 2}{4}\\&\times \biggl [\max \biggl \{\biggl | \frac{\partial ^2f(a,c)}{\partial x\partial y}\biggl |, \biggl | \frac{\partial ^2f(a,d)}{\partial x\partial y}\biggl |\biggl \} +\max \biggl \{\biggl | \frac{\partial ^2f(b,c)}{\partial x\partial y}\biggl |, \biggl | \frac{\partial ^2f(b,d)}{\partial x\partial y}\biggl |\biggl \}\biggl ]. \end{aligned}$$

### **Corollary 2**

*Under the conditions of Theorem* 4,

1. *If*$$s=0$$, *then*
  $$\begin{aligned} |\Phi (f;a,b,c,d)|\le & {} \frac{(b-a)(d-c)}{4}\biggl (\frac{1}{4}\biggr )^{1/q}\\&\times \biggl [\max \biggl \{\biggl | \frac{\partial ^2f(a,c)}{\partial x\partial y}\biggl |^q, \biggl | \frac{\partial ^2f(a,d)}{\partial x\partial y}\biggl |^q\biggl \} +\max \biggl \{\biggl | \frac{\partial ^2f(b,c)}{\partial x\partial y}\biggl |^q, \biggl | \frac{\partial ^2f(b,d)}{\partial x\partial y}\biggl |^q\biggl \}\biggl ]^{1/q}; \end{aligned}$$
2. *If*$$s=1$$, *then*
  $$\begin{aligned} |\Phi (f;a,b,c,d)|\le & {} \frac{(b-a)(d-c)}{8}\biggl (\frac{1}{12}\biggr )^{1/q}\\&\times \Bigl \{\bigl [M_q(a,c,d)+2M_q(b,c,d)\bigl ]^{1/q} +\bigl [2M_q(a,c,d) +M_q(b,c,d)\bigl ]^{1/q}\Bigl \}. \end{aligned}$$

### **Theorem 5**

*Let*$f:\Delta =[a,b]\times [c,d]\subseteq \mathbb {R}^2\rightarrow \mathbb {R}$*have partial derivatives and*$\frac{\partial ^2f}{\partial x\partial y}\in L_1(\Delta )$. *If*$\bigl | \frac{\partial ^2f}{\partial x\partial y}\bigr |^q$*is an* (*s*, QC)-*convex function on the co-ordinates on*$\Delta$*with*$a<b$*and*$c<d$*for some*$s \in [-1,1]$, $q>1$, *and*$0\le \ell \le q$, *then*

1. When $$s \in (-1,1]$$,
  $$\begin{aligned} |\Phi (f;a,b,c,d)|&\le \frac{(b-a)(d-c)}{16}\biggl (\frac{q-1}{2q-\ell -1}\biggr )^{1-1/q} \biggl (\frac{1}{2^{s-1}(\ell +1)(s+1)(s+2)}\biggr )^{1/q}\\&\quad \times \Bigl \{\bigl [(s+1)M_q(a,c,d)+(2^{s+2}-s-3)M_q(b,c,d)\bigl ]^{1/q}\\&\quad +\bigl [(2^{s+2}-s-3)M_q(a,c,d) +(s+1)M_q(b,c,d)\bigl ]^{1/q}\Bigl \}; \end{aligned}$$
2. *When*$$s=-1$$,
  $$\begin{aligned} |\Phi (f;a,b,c,d)|\le & {} \frac{(b-a)(d-c)}{16}\biggl (\frac{q-1}{2q-\ell -1}\biggr )^{1-1/q} \biggl (\frac{4}{\ell +1}\biggr )^{1/q}\Bigl \{\bigl [M_q(a,c,d)\\&+(2\ln 2-1)M_q(b,c,d)\bigl ]^{1/q} +\bigl [(2\ln 2-1)M_q(a,c,d) +M_q(b,c,d)\bigr ]^{1/q}\Bigl \}, \end{aligned}$$

*where*$M_q(u,c,d)$*is defined by* (19).

### *Proof*

If $s \in (-1,1]$, similar to the proof of the inequality (17), we can acquire

$$\begin{aligned} |\Phi&(f;a,b,c,d)| \\&\le (b-a)(d-c)\biggl \{\biggl (\int _0^{1/2}\int _0^{1/2}t\lambda ^{(q-\ell )/(q-1)}\mathrm{d}t\mathrm{d}\lambda \biggr )^{1-1/q}\\&\quad \times \biggl [\int _0^{1/2}\int _0^{1/2}t\lambda ^\ell \biggl | \frac{\partial ^2}{\partial x\partial y} f(ta+(1-t)b,\lambda c+(1-\lambda )d)\biggr |^q\mathrm{d}t\mathrm{d}\lambda \biggl ]^{1/q}\\&\quad +\biggl ( \int _{1/2}^1\int _0^{1/2}t(1-\lambda )^{(q-\ell )/(q-1)}\mathrm{d}t\mathrm{d}\lambda \biggr )^{1-1/q}\\&\quad \times \biggl [ \int _{1/2}^1\int _0^{1/2}t(1-\lambda )^\ell \biggl | \frac{\partial ^2}{\partial x\partial y} f(ta+(1-t)b,\lambda c+(1-\lambda )d)\biggr |^q\mathrm{d}t\mathrm{d}\lambda \biggl ]^{1/q}\\&\quad +\biggl (\int _0^{1/2}\int _{1/2}^1(1-t)\lambda ^{(q-\ell )/(q-1)}\mathrm{d}t\mathrm{d}\lambda \biggr )^{1-1/q}\\&\quad \times \biggl [\int _0^{1/2}\int _{1/2}^1(1-t)\lambda ^\ell \biggl | \frac{\partial ^2}{\partial x\partial y} f(ta+(1-t)b,\lambda c+(1-\lambda )d)\biggr |^q\mathrm{d}t\mathrm{d}\lambda \biggl ]^{1/q}\\&\quad +\biggl (\int _{1/2}^1\int _{1/2}^1(1-t)(1-\lambda )^{(q-\ell )/(q-1)}\mathrm{d}t\mathrm{d}\lambda \biggr )^{1-1/q}\\&\quad \times \biggl [ \int _{1/2}^1\int _{1/2}^1(1-t)(1-\lambda )^\ell \biggl | \frac{\partial ^2}{\partial x\partial y} f(ta+(1-t)b,\lambda c+(1-\lambda )d)\biggr |^q\mathrm{d}t\mathrm{d}\lambda \biggl ]^{1/q}\biggl \}\\&=(b-a)(d-c)\biggl [\frac{q-1}{8(2q-\ell -1)}\biggl (\frac{1}{2}\biggr )^{(2q-\ell -1)/(q-1)}\biggr ]^{1-1/q}\\&\quad \times \biggl \{ \biggl [\int _0^{1/2}\int _0^{1/2}t\lambda ^\ell \biggl | \frac{\partial ^2}{\partial x\partial y} f(ta+(1-t)b,\lambda c+(1-\lambda )d)\biggr |^q\mathrm{d}t\mathrm{d}\lambda \biggl ]^{1/q}\\&\quad +\biggl [ \int _{1/2}^1\int _0^{1/2}t(1-\lambda )^\ell \biggl | \frac{\partial ^2}{\partial x\partial y} f(ta+(1-t)b,\lambda c+(1-\lambda )d)\biggr |^q\mathrm{d}t\mathrm{d}\lambda \biggl ]^{1/q}\\&\quad +\biggl [\int _0^{1/2}\int _{1/2}^1(1-t)\lambda ^\ell \biggl | \frac{\partial ^2}{\partial x\partial y} f(ta+(1-t)b,\lambda c+(1-\lambda )d)\biggr |^q\mathrm{d}t\mathrm{d}\lambda \biggl ]^{1/q}\\&\quad +\biggl [\int _{1/2}^1\int _{1/2}^1(1-t)(1-\lambda )^\ell \biggl | \frac{\partial ^2}{\partial x\partial y} f(ta+(1-t)b,\lambda c+(1-\lambda )d)\biggr |^q\mathrm{d}t\mathrm{d}\lambda \biggl ]^{1/q}\biggl \}\\&\le (b-a)(d-c)\biggl (\frac{q-1}{2q-\ell -1}\biggr )^{1-1/q} \biggl (\frac{1}{2}\biggr )^{(5q-\ell -4)/q}\\&\quad \times \biggl \{ 2\biggl [\frac{1}{2^{s+\ell +3}(s+1)(s+2)(\ell +1)}\bigl [(s+1)M_q(a,c,d)+(2^{s+2}-s-3)M_q(b,c,d)\biggl ]^{1/q}\\&\quad +2\biggl [\frac{1}{2^{s+\ell +3}(s+1)(s+2)(\ell +1)}\bigl [(2^{s+2}-s-3)M_q(a,c,d) +(s+1)M_q(b,c,d)\bigr ]\biggl ]^{1/q}\biggl \}\\&=\frac{(b-a)(d-c)}{16}\biggl (\frac{q-1}{2q-\ell -1}\biggr )^{1-1/q} \biggl (\frac{1}{2^{s-1}(\ell +1)(s+1)(s+2)}\biggr )^{1/q}\\&\quad \times \Bigl \{\bigl [(s+1)M_q(a,c,d)+(2^{s+2}-s-3)M_q(b,c,d)\bigl ]^{1/q}\\&\quad +\bigl [(2^{s+2}-s-3)M_q(a,c,d) +(s+1)M_q(b,c,d)\bigl ]^{1/q}\Bigl \}. \end{aligned}$$

If $s=-1$, similarly one can see that

$$\begin{aligned}&|\Phi (f;a,b,c,d)| \le (b-a)(d-c)\biggl (\frac{q-1}{2q-\ell -1}\biggr )^{1-1/q} \biggl (\frac{1}{2}\biggr )^{(5q-\ell -4)/q}\\&\qquad \times \biggl \{ \biggl [\int _0^{1/2}\int _0^{1/2}t\lambda ^\ell \biggl | \frac{\partial ^2}{\partial x\partial y} f(ta+(1-t)b,\lambda c+(1-\lambda )d)\biggr |^q\mathrm{d}t\mathrm{d}\lambda \biggl ]^{1/q}\\&\qquad +\biggl [ \int _{1/2}^1\int _0^{1/2}t(1-\lambda )^\ell \biggl | \frac{\partial ^2}{\partial x\partial y} f(ta+(1-t)b,\lambda c+(1-\lambda )d)\biggr |^q\mathrm{d}t\mathrm{d}\lambda \biggl ]^{1/q}\\&\qquad +\biggl [\int _0^{1/2}\int _{1/2}^1(1-t)\lambda ^\ell \biggl | \frac{\partial ^2}{\partial x\partial y} f(ta+(1-t)b,\lambda c+(1-\lambda )d)\biggr |^q\mathrm{d}t\mathrm{d}\lambda \biggl ]^{1/q}\\&\qquad +\biggl [ \int _{1/2}^1\int _{1/2}^1(1-t)(1-\lambda )^\ell \biggl | \frac{\partial ^2}{\partial x\partial y} f(ta+(1-t)b,\lambda c+(1-\lambda )d)\biggr |^q\mathrm{d}t\mathrm{d}\lambda \biggl ]^{1/q}\biggl \}\\&\quad \le (b-a)(d-c)\biggl (\frac{q-1}{2q-\ell -1}\biggr )^{1-1/q} \biggl (\frac{1}{2}\biggr )^{(5q-\ell -4)/q}\\&\qquad \times \biggl \{ 2\biggl [\frac{1}{2^{\ell +2}(\ell +1)} \bigl [M_q(a,c,d)+(2\ln 2-1)M_q(b,c,d)\bigl ]\biggl ]^{1/q}\\&\qquad +2\biggl [\frac{1}{2^{\ell +2}(\ell +1)}\bigl [(2\ln 2-1)M_q(a,c,d) +M_q(b,c,d)\bigr ]\biggl ]^{1/q}\biggl \}\\&\quad =\frac{(b-a)(d-c)}{16}\biggl (\frac{q-1}{2q-\ell -1}\biggr )^{1-1/q} \biggl (\frac{4}{\ell +1}\biggr )^{1/q}\\&\qquad \times \Bigl \{\bigl [M_q(a,c,d)+(2\ln 2-1)M_q(b,c,d)\bigl ]^{1/q}\\&\qquad +\bigl [(2\ln 2-1)M_q(a,c,d) +M_q(b,c,d)\bigr ]^{1/q}\Bigl \}. \end{aligned}$$

The proof of Theorem 5 is complete.$\square$

### **Corollary 3**

*Under the conditions of Theorem* 5, *when*$\ell =1$,

1. *If*$$s \in (-1,1]$$, *then*
  $$\begin{aligned} |\Phi (f;a,b,c,d)|&\le \frac{(b-a)(d-c)}{32} \biggl (\frac{1}{2^{s-1}(s+1)(s+2)}\biggr )^{1/q}\\&\quad \times \Bigl \{\bigl [(s+1)M_q(a,c,d)+(2^{s+2}-s-3)M_q(b,c,d)\bigl ]^{1/q}\\&\quad +\bigl [(2^{s+2}-s-3)M_q(a,c,d) +(s+1)M_q(b,c,d)\bigl ]^{1/q}\Bigl \}; \end{aligned}$$
2. *if*$$s=-1$$, *then*
  $$\begin{aligned} |\Phi (f;a,b,c,d)|\le & {} \frac{(b-a)(d-c)2^{2/q}}{32}\\&\times \Bigl \{\bigl [M_q(a,c,d)+(2\ln 2-1)M_q(b,c,d)\bigl ]^{1/q} +\bigl [(2\ln 2-1)M_q(a,c,d) +M_q(b,c,d)\bigr ]^{1/q}\Bigl \}. \end{aligned}$$

### **Corollary 4**

*Under the conditions of Theorem* 5, *when*$\ell =q$,

1. *If*$$s \in (-1,1]$$, *then*
  $$\begin{aligned} |\Phi (f;a,b,c,d)|&\le \frac{(b-a)(d-c)}{16} \biggl (\frac{1}{2^{s-1}(q+1)(s+1)(s+2)}\biggr )^{1/q}\\&\quad \times \Bigl \{\bigl [(s+1)M_q(a,c,d)+(2^{s+2}-s-3)M_q(b,c,d)\bigl ]^{1/q}\\&\quad +\bigl [(2^{s+2}-s-3)M_q(a,c,d) +(s+1)M_q(b,c,d)\bigl ]^{1/q}\Bigl \}; \end{aligned}$$
2. *If*$$s=-1$$, *then*
  $$\begin{aligned}&|\Phi (f;a,b,c,d)| \le \frac{(b-a)(d-c)}{16} \biggl (\frac{4}{q+1}\biggr )^{1/q}\\&\quad \times \Bigl \{\bigl [M_q(a,c,d)+(2\ln 2-1)M_q(b,c,d)\bigl ]^{1/q} +\bigl [(2\ln 2-1)M_q(a,c,d) +M_q(b,c,d)\bigr ]^{1/q}\Bigl \}. \end{aligned}$$

### **Theorem 6**

*Let*$f:\Delta =[a,b]\times [c,d]\subseteq \mathbb {R}^2\rightarrow \mathbb {R}$*have partial derivatives and*$\frac{\partial ^2f}{\partial x\partial y}\in L_1(\Delta )$. *If*$\bigl | \frac{\partial ^2f}{\partial x\partial y}\bigr |^q$*is an* (*s*, QC)-*convex function on the co-ordinates on*$\Delta$*with*$a<b$*and*$c<d$*for some*$s \in (-1,1]$*and*$q>1$, *then*

$$\begin{aligned} |\Phi (f;a,b,c,d)|\le & {} \frac{(b-a)(d-c)}{4}\biggl (\frac{q-1}{2q-1}\biggl )^{2(1-1/q)} \biggl (\frac{1}{s+1}\biggr )^{1/q}\\&\times \biggl [\max \biggl \{\biggl |\frac{\partial ^2 f(a,c)}{\partial x\partial y}\biggl |^q, \biggl | \frac{\partial ^2 f(a,d)}{\partial x\partial y}\biggl |^q\biggl \} +\max \biggl \{\biggl | \frac{\partial ^2f(b,c)}{\partial x\partial y}\biggl |^q, \biggl | \frac{\partial ^2f(b,d)}{\partial x\partial y}\biggl |^q\biggl \}\biggr ]^{1/q}. \end{aligned}$$

### *Proof*

From Lemma 1, Hölder’s integral inequality, the co-ordinated $(s, \text {QC})$-convexity of $\bigl | \frac{\partial ^2f}{\partial x\partial y}\bigr |^q$, and Lemma 2, it follows that

$$\begin{aligned}&|\Phi (f;a,b,c,d)| \\&\quad \le (b-a)(d-c)\biggl (\int _0^1\int _0^1|K(t,\lambda )|^{q/(q-1)}\mathrm{d}t\mathrm{d}\lambda \biggr )^{1-1/q}\\&\qquad \times \biggl [\int _0^1\int _0^1\biggl | \frac{\partial ^2}{\partial x\partial y} f(ta+(1-t)b,\lambda c+(1-\lambda )d)\biggr |^q\mathrm{d}t\mathrm{d}\lambda \biggl ]^{1/q}\\&\quad \le (b-a)(d-c)\biggl [\biggl (\frac{q-1}{2q-1}\biggl )^2\biggl (\frac{1}{4}\biggr )^{q/(q-1)}\biggr ]^{1-1/q}\\&\qquad \times \biggl \{\int _0^1\biggl [t^{s}\max \biggl \{\biggl |\frac{\partial ^2 f(a,c)}{\partial x\partial y}\biggl |^q, \biggl | \frac{\partial ^2 f(a,d)}{\partial x\partial y}\biggl |^q\biggl \} +(1-t)^{s}\max \biggl \{\biggl | \frac{\partial ^2f(b,c)}{\partial x\partial y}\biggl |^q, \biggl | \frac{\partial ^2f(b,d)}{\partial x\partial y}\biggl |^q\biggl \} \biggr ]\mathrm{d}t \biggr \}^{1/q}\\&\quad =\frac{(b-a)(d-c)}{4}\biggl (\frac{q-1}{2q-1}\biggl )^{2(1-1/q)} \biggl (\frac{1}{s+1}\biggr )^{1/q}\\&\qquad \times \biggl [\max \biggl \{\biggl |\frac{\partial ^2 f(a,c)}{\partial x\partial y}\biggl |^q, \biggl | \frac{\partial ^2 f(a,d)}{\partial x\partial y}\biggl |^q\biggl \} +\max \biggl \{\biggl | \frac{\partial ^2f(b,c)}{\partial x\partial y}\biggl |^q, \biggl | \frac{\partial ^2f(b,d)}{\partial x\partial y}\biggl |^q\biggl \} \biggr ]^{1/q}. \end{aligned}$$

Theorem 6 is thus proved.$\square$

## Conclusions

Our main results in this paper are Definitions 11 to 12 and those integral inequalities of Hermite–Hadamard type in Theorems 4 to 6.
